# Mpox intra-action review in the Philippines, 2025

**DOI:** 10.5365/wpsar.2026.17.3.1338

**Published:** 2026-09-07

**Authors:** Yui Sekitani, Gloria Balboa, Rowena Capistrano, Judith Mariano, Almira Gatchalian, Liezl Fernandez, Anne Dominique Matuba, Anna Marie Celina Garfin, Jose Gerard Belimac, Angelica Mae Oira, Soonjong Bae, Princess Mhyco Esguerra, Cling Malaco, Kathleen E Ryan, Juan Paolo Tonolete, Kathleen Taylor Warren, Wenyajing Zhang, Sean T Casey

**Affiliations:** a World Health Organization Representative Office for the Philippines, Manila, Philippines.; b Department of Health, Manila, Philippines.; c World Health Organization Regional Office for the Western Pacific, Manila, Philippines.; d Department of Public Health and Health Policy, Graduate School of Biomedical and Health Sciences, Hiroshima University, Hiroshima, Japan.

The Director-General of the World Health Organization (WHO) twice declared mpox (previously monkeypox) a public health emergency of international concern (PHEIC): first in July 2022 (ended May 2023) and again in August 2024 (ended September 2025). (*1*, *2*) A PHEIC triggers temporary recommendations under the International Health Regulations (2005) to coordinate global response.

On 18 August 2024, the Philippine Department of Health (DOH) confirmed its 10th case of mpox, following earlier detections in 2022 and 2023. (*3*) In response, the DOH established the Interim Mpox Task Force through Department Personnel Order No. 2024–5521 to lead the overall national response. (*4*) The Task Force comprised representatives from DOH offices, WHO staff and members of the DOH Emerging and Re-emerging Infectious Diseases Strategic Advisory Group of Experts (EREID-SAGE), with expertise in dermatology, infectious diseases, social science and genomics. The Task Force developed and published the 2024 Philippine Mpox National Action Plan in September 2024. (*5*)

In March 2025, the DOH, in collaboration with the WHO Representative Office for the Philippines and the Regional Office for the Western Pacific, conducted the first intra-action review (IAR) of a national mpox response in the WHO Western Pacific Region. The objective was to identify strengths, challenges and lessons learned from the 2022–2024 response.

## PROCESS

In line with WHO guidance, the IAR (*6*) incorporated perspectives from national and regional health authorities, public health programme managers, emergency response teams, clinicians and affected communities. Participants were purposively selected to represent each of the six task groups of the Task Force: policy and planning, surveillance, risk communication and community engagement, access to biologics, clinical and public health management, and logistics and finance.

The IAR brought together a total of 69 participants, with the majority from the DOH Central Office, three from the Philippine National AIDS Council and the Food and Drug Administration, six from Centers for Health Development (regional health offices), two from a civil society organization (CSO) active in health interventions with key populations, and five EREID-SAGE members. Seven WHO staff served as facilitators. Data were gathered through focus group discussions structured around five questions adapted from the WHO IAR framework:

What was in place before the response?
What happened during the response?
What went well and what went less well?
What can be improved?
What are the ways forward?

No individual structured interviews were conducted. Instead, a World Cafe method was used, involving rounds of discussion across groups, with participants rotating and building on the inputs of others. Discussion outputs were grouped based on the functions of the task groups, and priority actions were agreed through consensus during plenary sessions. **Table 1** presents the key findings, challenges and priority actions.

**Table 1:** Key findings, challenges and priority actions by task group function, Philippines, 2025

Task group	Strengths/best practices	Challenges	Priority actions
**Policy and planning**	**Technical guidelines on surveillance, screening, case management and infection control were developed in response to early mpox cases in Africa.** **Technical consultations with EREID-SAGE and the HIV Technical Working Group supported evidence-informed and context-specific guidelines.**	**DOH operated through vertical programme structures with limited workforce capacity.** **There was no formal policy for immediate data-sharing with subnational offices. Data privacy concerns and stigma were mentioned to affect contact tracing and public trust.**	**Develop and issue a policy to integrate EREID mechanisms and systems across programmes.** **Formalize inter-bureau and external data-sharing agreements and protocols to institutionalize coordination and response mechanisms.**
**Surveillance**	**Prior investments had already been made in systems strengthening and workforce capacity, including procurement for testing and training on specimen collection, operationalization of specimen collection guidelines and the development of an internal real-time national dashboard.**	**There were challenges in contact tracing, data verification and case tagging, as well as limited testing outside Metro Manila during the initial phase of the response.** **Data-sharing and dashboard access were limited for local government units and external stakeholders.**	**Strengthen and standardize laboratory human resource requirements and expand capacity-building efforts at subnational levels through a training-of-trainers approach.** **Incorporate mpox testing into the National Laboratory Strategic Plan.** **Enhance the dashboard and identify a more sustainable and interoperable reporting platform.**
**Risk communication and community engagement**	**A multisectoral approach was adopted to raise public awareness, support timely response, and strengthen social listening and infodemic management. Social media, town halls, CSO partnerships and medical society collaboration helped reach target audiences.**	**There were challenges related to sustaining inclusive messaging and combating stigma, low public awareness and limited access to trained health-care providers and health promotion practitioners at the community level.**	**Engage CSOs, medical societies, development partners and community champions as primary messengers to deliver trusted, evidence-based, timely, audience-appropriate information.** **Use diverse communication platforms to better reach target audiences.**
**Access to biologics**	**Active engagement with regional and global partners, including ASEAN and international health platforms, facilitated early access to information on vaccine development and pooled procurement mechanisms.**	**Delays in regulatory authorization for ASEAN-procured mpox vaccines limited timely access.** **Existing policy restricted emergency use authorization to COVID-19 vaccines and medications only.**	**Review and update policies on the importation and management of foreign-donated health products.** **Advocate for a general authorization pathway for non-COVID-19 emerging/re-emerging disease vaccines.** **Reactivate the National Immunization Technical Advisory Group to provide evidence-based guidance on outbreak response immunization.**
**Clinical and public health management**	**An established patient navigation and referral mechanism ensured continuity of care.** **Dermatologists and infectious disease specialists improved care pathways, as many cases sought consultation in private or sexual health clinics.**	**There was limited knowledge among some health-care workers on clinical care and supportive management.**	**Develop a Philippine mpox case atlas to share information.** **Strengthen feedback mechanisms and provide training on detection and case management with continued support from medical societies.**
**Logistics and finance**	**Response activities were financed through existing budget lines with additional allocations for critical supplies.** **A digital information system provided near-real-time inventory tracking and strategic augmentation of supplies such as personal protective equipment.**	**While funding was available, access mechanisms needed improvement.** **Subnational coordinators had limited capacity in commodity quantification and forecasting.**	**Conduct table-top simulation exercises to test guidelines and operational readiness.** **Facilitate coordination between technical and logistics offices to develop forecasting tools and improve warehouse management.** **Advocate for pre-positioning and dedicated EREID response funds.**

ASEAN: Association of South-eastern Asian Nations; CSO: civil society organization; DOH: Department of Health; EREID: Emerging and Re-Emerging Infectious Diseases; SAGE: Strategic Advisory Group of Experts.

## Discussion

The mpox IAR included CSO representatives and scientific experts who may not typically be involved in national-level outbreak response coordination, broadening participation compared with the Philippines’ COVID-19 after-action review in 2023. (*7*) That review involved multiple agencies but no community representatives. CSO participants representing key affected populations highlighted gaps in coordination between the DOH, medical societies and other CSOs. Strengthening these links is important for reaching at-risk populations, disseminating accurate health information and improving early detection. (*8*)

Overall, best practices and strengths during the mpox response were largely identified as deriving from lessons learned during the COVID-19 pandemic response. Following the IAR conducted in March 2025, and in the context of the broader response, the Philippines developed an updated Mpox National Action Plan for 2026–2028 and the TF structure. The establishment of the TF was identified as one of the key best practices, as it ensured a well coordinated and organized response effort through regular meetings and sustained engagement among members. This coordinated mechanism also facilitated the country’s capability to mobilize access to medical countermeasures. In December 2024, the Philippines accessed Tecovirimat through the pooled procurement mechanism of the Association of South-eastern Asian Nations (ASEAN). (*9*) Using available regulatory pathways including a compassionate special permit, a total of 160 full treatment courses were authorized and allocated to several DOH-designated hospitals. In February 2026, the Philippines also secured 2220 doses of live, non-replicating smallpox and mpox vaccine (MVA-BN, brand name JYNNEOS^®^) through the same ASEAN procurement channel.

The IAR was conducted during an ongoing health emergency, enabling real-time adjustments and continuous learning. (*10*) During the COVID-19 pandemic, an IAR identified similar coordination and data-sharing gaps across government agencies, suggesting these are systemic challenges and are not unique to the mpox context. Although mpox had faded from the headlines, the IAR provided an opportunity to help the DOH identify areas for improvement that are often overlooked in routine operations. It also promoted broader participation in health emergency responses. As emphasized by the mpox IAR, responses to dynamic and complex outbreaks require stronger coordination and more timely, interoperable data-sharing. It also highlighted the importance of adapting response strategies to at-risk populations, including leveraging existing systems such as HIV platforms, particularly for risk communication and community engagement, to ensure more inclusive and effective outreach.

This review had limitations. Although it brought together national and regional DOH offices, CSO representatives and scientific experts, providing a broad view of the mpox response, no local government units (LGUs) were represented. Given the role of LGUs in the decentralized Philippine health system, future IARs may further strengthen this approach by directly capturing LGU-level implementation experiences. Findings were based on self-reported experiences and may reflect recall and group discussion dynamics. The review did not include outcome data, such as case detection times or changes in transmission patterns, to measure the effect of the IAR on response performance. Thus, its findings may not be generalizable to other country contexts.

In conclusion, the Philippines’ experience shows that inclusive, participatory IARs can bring together diverse groups to identify and review priorities, thereby strengthening coordination, communication and collaboration to improve response efforts. Importantly, the mpox IAR helped institutionalize the use of IARs as a regular component of emergency preparedness and response in the Philippines. Recognizing their value in improving multisectoral coordination, the Government has incorporated IARs as one of the key response strategies in the current National Action Plan for Health Security, which was launched in May 2026, as well as the Emerging and Re-Emerging Infectious Diseases Strategic Plan 2026–2030. This integration reflects a broader commitment to systematically applying IARs to future public health threats and to enhancing public health responses during emergencies.
